# The Mouth

**Published:** 1881-07

**Authors:** 


### THE MOUTH.

The mouth, though a small thing, has something to do with the beauty of man; for every one has a pretty mouth though other features be deformed. There are exceptions to this I know. A learned Englishman has taken the pains to prepare statistics among thirty millions of inhabitants; he has found the mouths of three million subjects, which may be called truly ugly. You will agree that is not very many.

The lip thickened in just proportion is generally thought better. It increases the beauty and decreases the deformity. The thin lip has, I know not what, of dryness and doggedness which displeases one. And I have remarked that upon fifteen pretty faces with *thin lip*, ten of them had the appearance of being faces of wax.

They say there are a greater number of large than small mouths. A very large mouth is generally an indication of sulleness; a very small one of meanness. The grimaces at the corner of the mouth are indicative of malice, irony, and also of playfulness. A truly beautiful woman is endowed with a regular mouth, neither large nor small, with turgid lips, partly opened and ruddy. It is easier to find a beautiful woman with a well-proportioned large mouth, than with a small one. The little mouth is only the appendage of little women.

She who bites her lips is a woman who has neither fortune nor good luck in the world; she whose upper lip is covered with a light brown down, loves her neighbor very much; she who has a mouth somewhat irregular is of a gentle character and easy manners. And she who holds the mouth always opened has beautiful teeth, or she is wicked.

Almost every eloquent man has a thick lip. Biting the lip is an indication of sensibility and of affectation.

Stammering people have shrivelled, poor and very thin lips. Mutes, on the contrary, have mouths rarely wrinkled or deformed.

The very largest lips belong to the negroes of Dahomey and Gabon; the finest type to the Anglo-Saxon race. The very smallest mouth belongs to the Chinaman; the largest is that of the Samoyedes and Laplander.

Already, not very many years since, they began to make jaws and tongues as they make teeth. These artificial tongues are made of gutta-percha. They move about, taste, and they will also spit or spatter. They will likewise perform a number of other tasks, yet, with them one *can not talk.* Yet Macrobe declares that he saw distinctly a gladiator who had only a stump (or little piece) of a tongue, Eutienne of Byzance, has compared the mouth of a slanderer to the opening of a filthy place. But if the comparison is made, we should make it when the person blows his or her nose.—*Translated from Belgian Lancet.*

